# Concussion Assessment With Smartglasses: Validation Study of Balance Measurement Toward a Lightweight, Multimodal, Field-Ready Platform

**DOI:** 10.2196/mhealth.8478

**Published:** 2018-01-23

**Authors:** Joseph P Salisbury, Neha U Keshav, Anthony D Sossong, Ned T Sahin

**Affiliations:** ^1^ Neural Sensing and Biometrics Division TIAX LLC Lexington, MA United States; ^2^ Empowerment Lab Brain Power, LLC Cambridge, MA United States; ^3^ Department of Psychiatry Massachusetts General Hospital Boston, MA United States; ^4^ Harvard Medical School Boston, MA United States; ^5^ Department of Psychology Harvard University Cambridge, MA United States

**Keywords:** postural balance, wearable technology, accelerometry, mild traumatic brain injury

## Abstract

**Background:**

Lightweight and portable devices that objectively measure concussion-related impairments could improve injury detection and critical decision-making in contact sports and the military, where brain injuries commonly occur but remain underreported. Current standard assessments often rely heavily on subjective methods such as symptom self-reporting. Head-mounted wearables, such as smartglasses, provide an emerging platform for consideration that could deliver the range of assessments necessary to develop a rapid and objective screen for brain injury. Standing balance assessment, one parameter that may inform a concussion diagnosis, could theoretically be performed quantitatively using current off-the-shelf smartglasses with an internal accelerometer. However, the validity of balance measurement using smartglasses has not been investigated.

**Objective:**

This study aimed to perform preliminary validation of a smartglasses-based balance accelerometer measure (BAM) compared with the well-described and characterized waist-based BAM.

**Methods:**

Forty-two healthy individuals (26 male, 16 female; mean age 23.8 [SD 5.2] years) participated in the study. Following the BAM protocol, each subject performed 2 trials of 6 balance stances while accelerometer and gyroscope data were recorded from smartglasses (Glass Explorer Edition). Test-retest reliability and correlation were determined relative to waist-based BAM as used in the National Institutes of Health’s Standing Balance Toolbox.

**Results:**

Balance measurements obtained using a head-mounted wearable were highly correlated with those obtained through a waist-mounted accelerometer (Spearman rho, ρ=.85). Test-retest reliability was high (intraclass correlation coefficient, ICC_2,1_=0.85, 95% CI 0.81-0.88) and in good agreement with waist balance measurements (ICC_2,1_=0.84, 95% CI 0.80-0.88). Considering the normalized path length magnitude across all 3 axes improved interdevice correlation (ρ=.90) while maintaining test-retest reliability (ICC_2,1_=0.87, 95% CI 0.83-0.90). All subjects successfully completed the study, demonstrating the feasibility of using a head-mounted wearable to assess balance in a healthy population.

**Conclusions:**

Balance measurements derived from the smartglasses-based accelerometer were consistent with those obtained using a waist-mounted accelerometer. Additional research is necessary to determine to what extent smartglasses-based accelerometry measures can detect balance dysfunction associated with concussion. However, given the potential for smartglasses to perform additional concussion-related assessments in an integrated, wearable platform, continued development and validation of a smartglasses-based balance assessment is warranted. This approach could lead to a wearable platform for real-time assessment of concussion-related impairments that could be further augmented with telemedicine capabilities to integrate professional clinical guidance. Smartglasses may be superior to fully immersive virtual reality headsets for this application, given their lighter weight and reduced likelihood of potential safety concerns.

## Introduction

### Background

Mild traumatic brain injury (mTBI), also known as concussion, is a common injury in both sports, with an estimated annual incidence of 1.6-3.8 million in the United States alone [1], and modern war, with 297,478 diagnoses in US service members between 2000 and 2016 [2]. Prompt identification of a concussed individual and removal from activity is the most effective method to facilitate rapid recovery immediately following injury [3-6]. Unrecognized and untreated mTBI can put athletes and service members at greater risk for more substantial TBI, as well as chronic encephalopathy, later [7-10]. Unfortunately, failure to detect concussions in a timely fashion is common in both the sporting arena [11,12] and military [13], as the immediate symptoms can be subtle and difficult to detect.

Concussion is considered one of the most complex injuries in sports medicine to diagnose, assess, and manage [14]. Accurate diagnosis and recovery monitoring of concussion is further complicated as recommended assessments, including the Standardized Concussion Assessment Tool (SCAT) and Military Acute Concussion Evaluation (MACE), rely heavily on patient symptom self-reporting [15]. Concussions can escape detection in committed athletes who are motivated to remain in the game [16], which further highlights the need for unbiased and objective sideline assessments [17]. In a military setting, service members who experience concussions are frequently under severe levels of physiological and emotional stress and may be unable to recognize or recall symptoms [13]. Injuries are frequently embedded in longer, continuous missions, where removing oneself from active combat to report a mild injury often does not occur [13]. Furthermore, concussion assessments commonly used in these settings, including MACE, lack diagnostic utility as early as 12 hours after injury [18]. Thus, improved, more objective methods for detection and recovery monitoring following concussions are a priority for both athletic organizations [19-21] and US Department of Defense health care providers [22-24].

### Approach

Concussion diagnosis and recovery monitoring requires a multifaceted and multimodal approach [25]. Concussion results in a range of clinical signs and symptoms, including impaired movement, balance, oculomotor function, attention, memory, and emotional functioning [26]. Unbiased and objective assessments of reaction time, balance, oculomotor function, and heart rate variability using an automated, digitized platform could substantially enhance the field recognition of concussion [25,27]. Although any single measure may not be precise enough to confidently diagnose concussion, a standardized combination of these measures could produce a sufficient concussion diagnostic metric. A lightweight and portable tool combining appropriate measures in a rapid assessment battery would be useful in both contact sports and the battlefield, where fast-paced and disorganized environments often obscure incidents of injury [25,28].

Considering the variety of assessments necessary, we examined whether smartglasses, an emerging computing platform, could be leveraged to provide a lightweight, portable, and wearable solution for measuring concussion-related impairments. Smartglasses, such as Glass (Google/X, Mountain View, CA), typically have a built-in 9-axis inertial measurement unit (IMU) that includes a 3-axis accelerometer, along with a gyroscope and magnetometer. Accelerometer-based balance assessments have garnered increased attention because of the widespread availability of accelerometers as a component of consumer smartphones [29,30]. The balance accelerometer measure (BAM) was developed as part of the National Institutes of Health’s (NIH) Standing Balance Toolbox to provide a low-cost assessment [31], which can be administered through the use of an iOS app. Likewise, the Sway balance app [32-36] for iOS was designed to provide an easily accessible method for quantitative balance assessment and has obtained FDA (Food and Drug Administration) clearance.

Smartglasses could enable self-administered balance assessments, as well as rehabilitative feedback, by providing real-time audio/visual instruction to the user while monitoring balance via the IMU. Although balance would only be one component of a concussion diagnostic metric, smartglasses could also deliver other relevant assessments, including vestibulo-ocular and cognitive assessments. Smartglasses could also serve as a processing hub for integration with other wearable sensors, including wearable electrophysiological devices. Finally, smartglasses could enable remote/telemedicine concussion diagnosis. A medical professional could receive data from the wearable sensor platform while communicating in real time with the injured or some untrained personnel to determine the need for further care [37].

### Goal of This Study

In this report, we sought to determine to what extent smartglasses-based balance measurement corresponded with a consumer smartphone attached at the waist, as in the NIH Toolbox Standing Balance Test. The objective of this study was to demonstrate the feasibility of obtaining quantitative balance measurements with smartglasses. These results could motivate future research in how smartglasses may be used to measure balance dysfunction and other concussion-related impairments. Although there exist multiple static balance protocols, the NIH Toolbox Standing Balance Test stances were used in this proof-of-concept study, given the availability of detailed methods and reference data available [31,38,39] for comparison between devices.

## Methods

### Subjects

A total of 42 individuals participated in this study (Table 1). Procedures were approved by the Asentral, Inc. Institutional Review Board (Newburyport, MA, USA) and the US Army Human Research Protection Office. Subjects were recruited from the public. An informed consent form describing the nature of the study, as well as the exclusion criteria, was completed by all participants.

Participants were required to be between the ages of 18 and 39 years, weigh no more than 250 pounds, and possess normal hearing and normal or corrected-to-normal vision. Each of these criteria was confirmed by participant self-report. Participants were excluded if they reported any preexisting condition that may alter their ability to balance normally. A set of specific conditions that could affect balance were described for participants. Specific conditions listed for participants included multiple sclerosis, Parkinson’s disease, Huntington’s disease, other movement disorders, stroke, cervical spine or physical mobility issues, more than 1 fall in the past 6 months not as a result of an accident, current pregnancy, dizziness or vertigo, any lower extremity injury that required medical attention in the last 3 months, and any surgeries within the last year. All participants attested they were not taking any medication to lower blood pressure or to control a heart problem. All participants also attested they were not under instruction by a supervising physician to avoid full/unrestricted physical activity. Individuals were also screened based on self-report for history of a diagnosed seizure disorder (or any seizures within the last 3 years), as well as extreme sensory sensitivity. All participants also attested to having no diagnosed macular degeneration, glaucoma, or cataracts, or any chronic or acute conditions resulting in pain, including diabetes or a history of joint replacement.

### Experimental Setup

Before administering the BAM protocol, subjects were outfitted with a gait belt. An Android smartphone (Samsung Galaxy S5, Samsung Galaxy S6, or LG Electronics/Google Nexus 5) was attached to the gait belt using a protective case with clip. The smartphone was attached upright, with the screen facing away from the subject. The subject was also given a pair of Glass Explorer Edition (henceforth, Glass) by the facilitator to wear. Subjects who normally wore glasses were given the option to wear Glass over or without their regular glasses. Subjects were asked to read a sentence on the display screen to confirm the screen was adjusted properly. A test exercise was administered on Glass to ensure subjects could (1) operate Glass by tapping on the side and (2) could hear a tone played from Glass.

**Figure 1:**
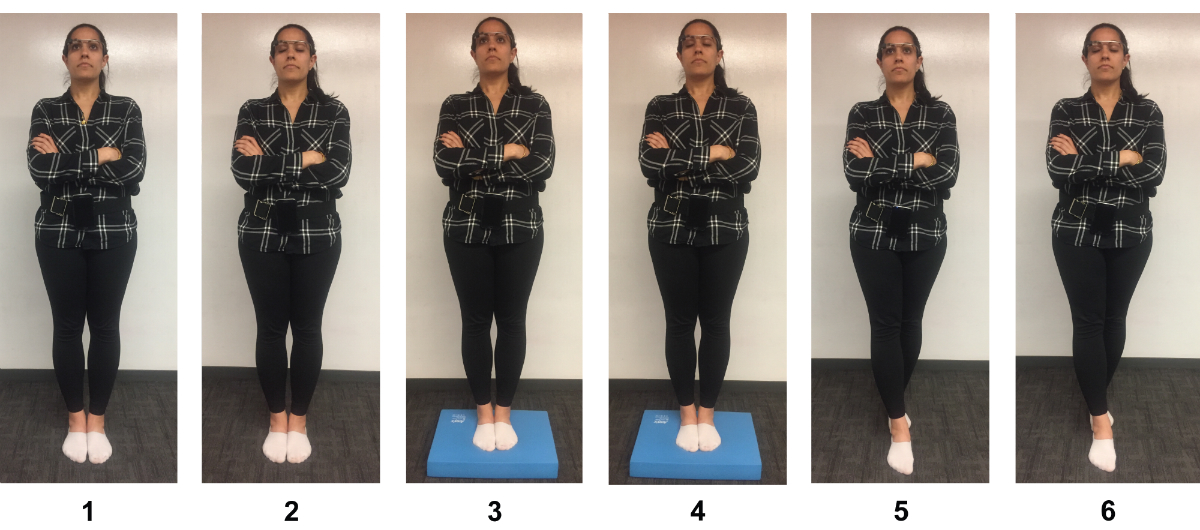
Balance accelerometer measure (BAM) protocol conditions. (1, 2) Feet together on a firm surface used for conditions 1 (eyes open) and 2 (eyes closed). (3, 4) Feet together on a foam surface used for conditions 3 (eyes open) and 4 (eyes closed). (5, 6) Feet in tandem stance on a firm surface used for conditions 5 (eyes open) and 6 (eyes closed).

The BAM protocol was administered as previously described [39]. The BAM protocol includes 6 standing conditions: (1) solid surface, feet together, eyes open, and (2) eyes closed; (3) foam surface (Airex Balance Pad, Specialty Foams, Switzerland), feet together, eyes open, and (4) eyes closed; (5) solid surface, tandem standing, eyes open, and (6) eyes closed (Figure 1). During each stance, all subjects were asked to stand quietly for 60 seconds and to look (in eyes-open conditions) at a symbol placed centrally at eye level 1 m from the subject. Subjects were instructed by the facilitator regarding stance following the instructions adapted from the NIH Toolbox Standing Balance Test [31,40]. Stance was also described on the smartglasses display screen. Subjects initiated each set of data collection by tapping the side of the smartglasses. A timer was displayed on Glass showing time remaining and a tone was played at the end of each timed stance. All subjects completed 2 attempts of all stances on the same day.

A trained study facilitator was present during the study and was ready to prevent the participant from falling. The study facilitator observed participants for failure to hold the demonstrated pose. Failures were recorded if (1) participant’s arms came off his/her chest, (2) participant’s knees bent, (3) participant’s feet moved out of original position (move or swivel out or are lifted), (4) participant bent forward at the waist (more than 45°), (5) participant opened his/her eyes during an eye-closed pose, or (6) participant says something like “I cannot do that” or “I do not feel safe trying that.”

### Data Acquisition

**Figure 2:**
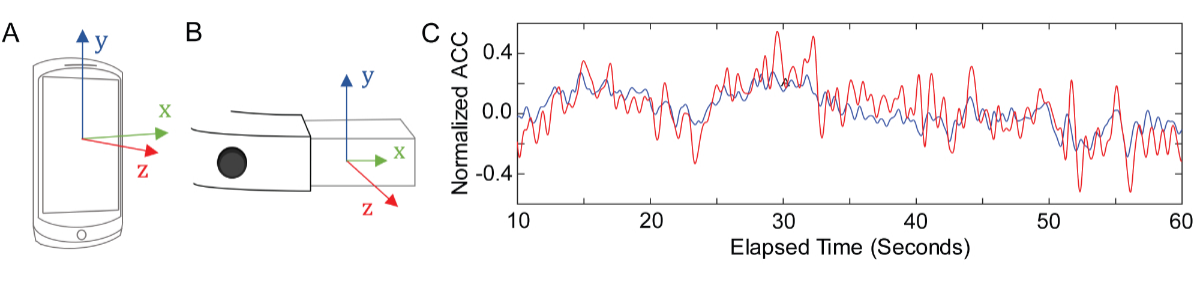
Accelerometer data (ACC) collection with smartphone and smartglasses. (A) Axes of accelerometer on Android smartphone compared with (B) Glass. (C) Example comparison of low-pass filtered ACC (z-axis) collected during a trial of condition 6 in Glass (red) compared with waist-mounted smartphone (blue).

An Android app was developed to synchronize recording of device IMU data between smartglasses and the waist-mounted smartphone (Figure 2). The app was installed on both Glass and the Android smartphones before testing. The app allowed Glass to pair with a smartphone via Bluetooth. Messages sent via Bluetooth from Google Glass to the smartphone were used to initiate a timer on Google Glass and begin storing IMU values (sampled at 50 Hz).

**Table 1:** Subject demographics (N=42).

Demographics	Mean (SD, range) or n (%)
Age in years, mean (SD, range)	23.8 (5.2, 18-37)
Gender, female, n (%)	16 (38)
Height in inches, mean (SD, range)	68 (3, 62-76)
Weight in pounds, mean (SD, range)	152 (32, 110-241)

Upon completion of each stance, time-stamped IMU data were saved on the device as a comma-delimited plain text file. When running on Google Glass, the app provides instructions on stance, a timer, and a tone that plays at the end of each stance session. At the end of each study session, data were transferred from respective devices to secure cloud storage.

### Data Analysis

Data files were imported into MATLAB 2016b (MathWorks, Native, MA, USA) for analysis, which included use of the Signal Processing Toolbox, the Statistics and Machine Learning Toolbox, and custom scripts. The first 10 seconds of data were discarded to ensure stability of measures (50 seconds of data total). Accelerometer data (ACC) from each trial was low-pass filtered using a fourth-order, Butterworth filter with a cutoff frequency of 1.25 Hz [39]. The normalized path length (NPL; mG/sec; higher values indicate more sway) of the anterior-posterior (AP) postural sway data was calculated as previously described [39]. NPL was also calculated from the combined ACC magnitude.

Trials recorded as failures by the study facilitator were excluded from further analysis. Smartglasses-based measurements of NPL along different axes were compared with smartphone measurements using Spearman rank correlation coefficient [41]. For comparison of differences between stances, the nonparametric Kruskal-Wallis test was used to compare mean ranks [42,43]. Normality of measurements within stance conditions was evaluated by the Anderson-Darling test [44]. Significant differences between correlation coefficients were determined by treating them as Pearson coefficients and using the standard Fisher z-transformation to compare using a standard normal procedure [45]. Test-retest reliability of NPL measurements was estimated for each condition between the 2 sessions by calculating the 2-way random, single-measure intraclass correlation coefficient, ICC_2,1_, and corresponding 95% CI [46,47]. NPL was standardized as previously described [39], and the composite score was calculated by adding together the standardized values across all 6 conditions.

## Results

### Measurement of Anterior-Posterior Sway with Smartglasses Correlates With Measurement at Waist

All 42 subjects successfully passed both trials on conditions 1 through 3, similar to previous reports [39]. Both trials of the eyes closed/foam surface condition (condition 4) were passed successfully by 37 subjects (88%). One subject failed a trial of the eyes-open/tandem stance condition (condition 5). Thirty subjects (71%) successfully passed 2 trials of the eyes closed/tandem stance condition (condition 6). All observed failures were recorded as feet moving out of the original position and/or arms coming off the chest. Overall, 2 trials on all 6 conditions were successfully passed by 28 subjects (66%).

**Figure 3:**
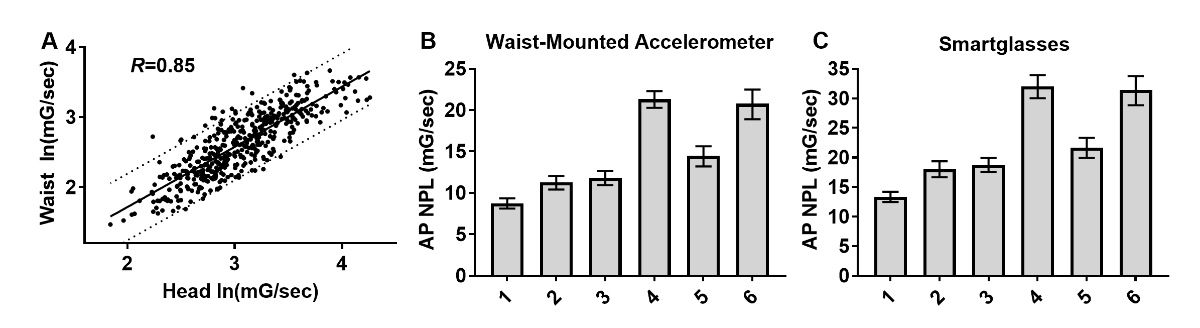
Anterior-posterior (AP) sway measured with smartglasses is highly correlated with waist-based accelerometry. (A) AP sway measured from the head was strongly correlated with AP sway measured from the waist (pooled data from all conditions with 95% prediction bands). Geometric mean and 95% CI for waist-based (B) and head-based (C) measurements of AP normalized path length (NPL) by condition.

NPL AP sway measured from the head was strongly correlated (Spearman rank correlation coefficient=.85) with NPL AP sway measured from the waist (Figure 3). Mean NPL AP sway measured from the waist was in good agreement with previously reported values [39], although we observed a higher mean for condition 6. The mean (SD) composite score was 21.4 (18.0), which was in good agreement with the previously reported value of 19.6 (15.3) for healthy subjects.

Although NPL measured from the head was generally larger than NPL measured from the waist in each trial, mean NPL AP sway measured from the head in each condition was observed to follow a similar trend as the means measured from the waist. Significant differences (Kruskal-Wallis, *P<*.001) were found between each set of eyes-open and eyes-closed conditions as well as between standing on feet together/firm surface compared with foam surface or tandem stance.

### Correlation Between Measurements Was Significantly Stronger When Calculating Normalized Path Length From All Three Axes

**Figure 4:**
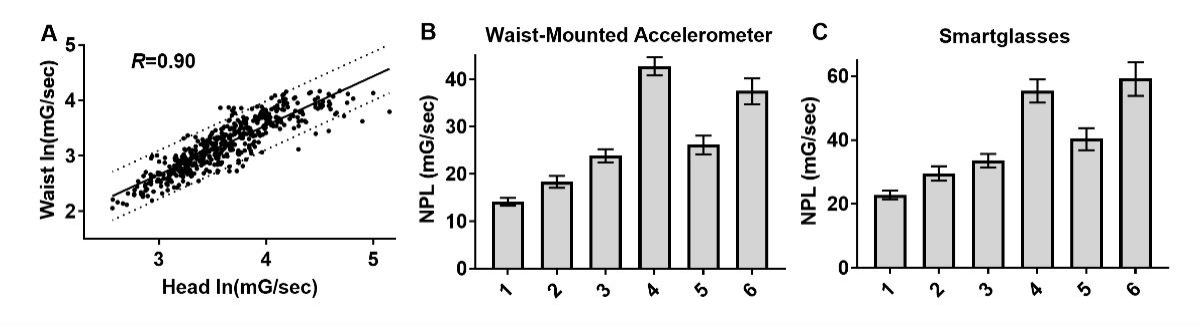
Total sway magnitude measured with smartglasses is highly correlated with waist-based accelerometry. (A) Total sway measured from head was more strongly correlated with sway measured from the waist. Geometric mean and 95% CI for waist-based (B) and head-based (C) measurement of total normalized path length (NPL) by condition.

Measuring sway along the ACC’s AP axis was previously shown to be sufficient to differentiate healthy subjects from subjects with vestibular disorders [39]. However, the additional ACC acquired from commercial off-the-shelf (COTS) smart devices may further enhance measurement accuracy, particularly along the mediolateral x-axis. Indeed, the NPL calculated using all 3 axes (total NPL) was found to have a significantly stronger correlation (Spearman rank correlation coefficient=.90, *P<*.001) between head- and waist-based measurements (Figure 4). Mean total NPL measured in each condition followed similar trends as using AP NPL only for both waist- and head-based measurements.

### Test-Retest Reliability of Measures Were Comparable Between Head and Waist

**Figure 5:**
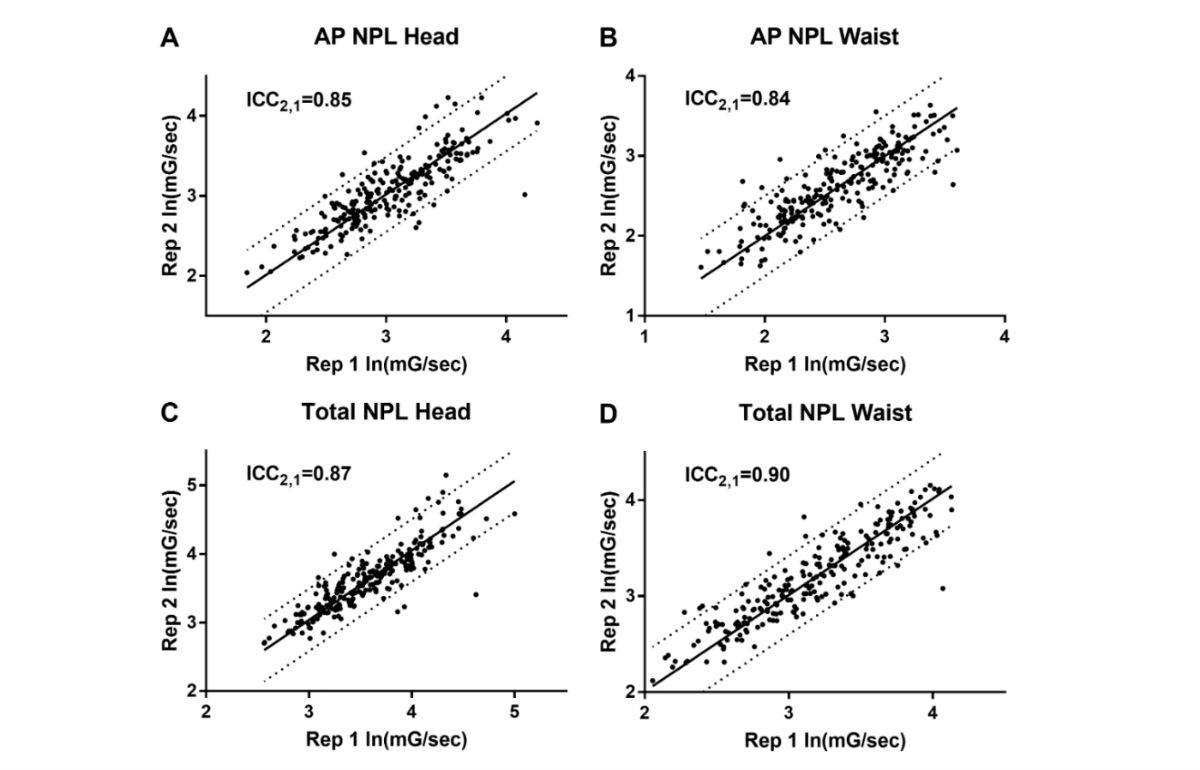
Head-mounted smartglasses have comparable test-retest reliability to waist-mounted balance accelerometer measurement. Test-retest intraclass correlation coefficient, ICC2,1, for accelerometer measures of postural sway along anterior-posterior (AP) (A=head, B=waist) and all axes (C=head, D=waist). NPL: normalized path length.

Previously, the test-retest reliability of NPL AP measured from the waist was found to be generally good (ICC≥0.74) across all conditions, except for condition 6 [39]. Here, same-day test-retest reliability of AP NPL measured from the head with smartglasses was found to be very good (Figure 5), with an ICC_2,1_ (95% CI) of 0.85 (0.81-0.88). This was comparable to our estimation of the test-retest reliability of waist-based AP NPL, which was 0.84 (0.80-0.88), agreeing with previously reported values.

Using total NPL, we found a slight improvement in test-retest reliability in both head- and waist-based measurements. ICC_2,1_ (95% CI) was found to be 0.87 (0.83-0.90) in the case of head-based measurement, as opposed to 0.90 (0.88-0.92) in the case of waist-based measurement.

## Discussion

### Principal Findings

To the best of our knowledge, this is the first study assessing the feasibility of using COTS smartglasses to perform quantitative standing balance measurement. This study indicates that head-based measurement of AP or total sway with smartglasses following the BAM protocol produces similar results to waist-based measurement. This included similar relative differences between test conditions as well as similar test-retest reliability. Condition effects observed with this protocol previously supported the validity of waist AP NPL as a measure of balance. Similar to these previous findings, sway measured with smartglasses was larger with eyes closed than with eyes open for all stance conditions. Sway was also larger in tandem stance and on foam surface conditions compared with corresponding conditions with feet together on a firm surface. These condition effects previously indicated that ACC measured from the waist was sensitive to changes in the sensory modalities available for balance, including vision and somatosensation [48]. In this study, we demonstrate that head-based ACC measurement using COTS smartglasses has a comparable sensitivity for measuring these differences.

### Comparison With Prior Work and Future Implications

The current wide availability of smartphones with IMU technology has made them an attractive platform to develop physical health assessments on. Along with standing balance, smartphone-based measurements are also being developed to objectively quantify a range of related functional mobility assessments [49-53]. Similarly, there are a growing number of dedicated wearables that have been developed to provide research- and clinical-grade balance, gait, full-body kinematics, and other functional mobility assessments. An important distinction between using the sensors in a smartphone compared with wearable hardware built for a specific function is that smartphones are already widely used by the population. Thus, smartphone-based assessments can be immediately accessible on this multifunctional platform. The multifunctional versatility of such a device could be particularly transformative in the battlefield, where there are practical limitations to amount of equipment that can be transported in various circumstances [28]. When considering the broader goal of having an objective balance assessment as only one component of a multifactorial concussion battery, neuroimaging and biomolecular assays could provide more definitive results and aid in differential diagnosis. However, the equipment needed to provide this level of certainty would be less practical for point-of-injury assessment and triage when a software-based assessment on a multipurpose device could sufficiently determine the need for additional care.

Leveraging the wide availability of COTS hardware to develop objective clinical assessments and rehabilitative strategies has motivated research into not just smartphones. The Nintendo Wii Balance Board [54-57] and Microsoft Kinect [58-60] are also being used in physical medicine. Indeed, there are now several FDA-cleared Kinect applications suitable for use in the clinic or at home that provide exercise guidance and remotely accessible patient performance metrics [61,62]. The sophistication of sensor-rich COTS hardware enables health care apps to be developed without the costs typically associated with dedicated health care equipment design, manufacturing, quality control, storage, distribution, etc. Admittedly, smartglasses are far from reaching the ubiquity of these devices. However, the market for smartglasses is projected to reach 3.4 million units by 2020, with health care being a major driver of smartglasses’ growth [63]. Smartglasses have been shown to be well tolerated in children and adults with autism spectrum disorder, providing evidence to support their use as an assistive device [64-66]. In the longer term, decline in costs, the solidification of applications and model features, and technology saturation of smartphone and tablet markets could push smartglasses to become a dominant consumer computing device [63]. It is this context, considering the potential future widespread availability of lightweight and portable head-mounted wearables, which motivates the research study described here.

**Figure 6:**
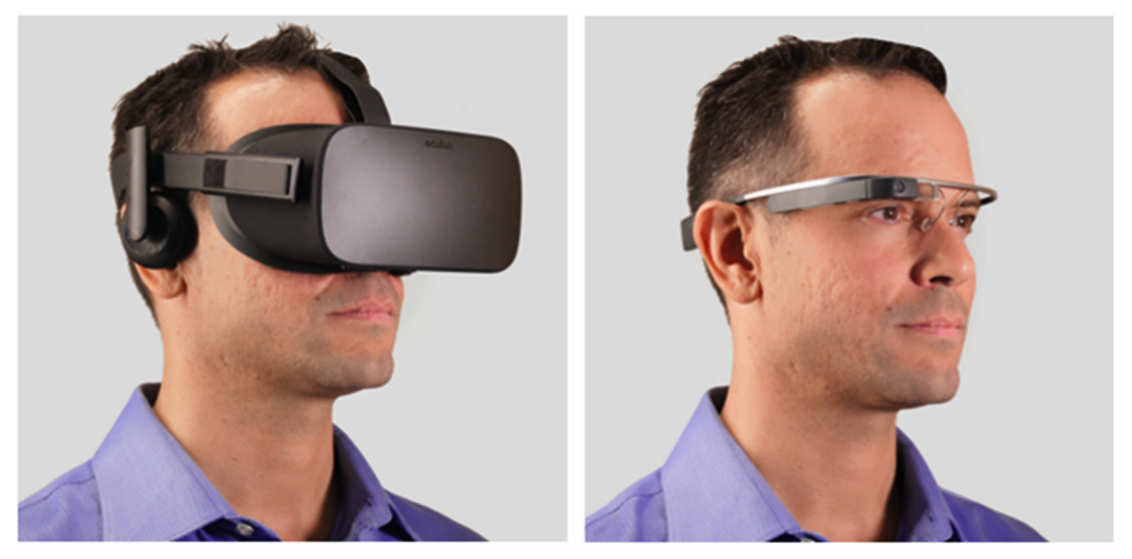
Fully immersive head-mounted device compared with partially immersive smartglasses. Fully immersive virtual reality headsets, such as Oculus Rift (left), completely block external stimuli, limiting their use in concussed populations where dizziness, nausea, and sensitivity to light are common persistent symptoms. Smartglasses, such as Glass (right), could provide a safer, more portable, and lighter-weight alternative. Of note, Glass weighs only 1.3 ounces—more than 10 times less than Oculus Rift.

Critically, it was not the objective of this study to determine whether smartglasses would provide a more sensitive measure than smartphones or act as a replacement for gold standard methods of clinical assessment. Rather, with this feasibility demonstrated, it can be discussed how smartglasses could have specific advantages over other COTS devices for assessment and rehabilitation of balance dysfunction related to brain injury. Recently, it was reported that a fully immersive head-mounted virtual reality (VR) system was successfully used to obtain repeatable balance assessment measurements in an elderly population [67]. Higher fall risk participants were found to change their tilt in the AP direction at a significantly higher rate. Although minimal simulator sickness was generally reported in this study, at least one participant dropped out of the study because of this issue. There were also significant differences in nausea pre- and postmeasurement. In terms of head-mounted wearables, smartglasses may be preferable to fully immersive VR headsets as they do not completely obscure external vision, which suggests they could be a safer alternative (Figure 6). Smartglasses could provide real-time feedback to correct balance instability during movement in an actual environment, such as through audio [68-70], and Glass has been shown to be feasible for external rhythmic cueing to improve gait in Parkinson’s patients [71]. Furthermore, fully immersive VR headsets often include foam that is pressed against the user’s face that can quickly become unsanitary, leading to hygiene concerns and the potential for disease transmission when used in a clinical setting [72]. Thus, smartglasses may be preferable in clinical use for not just balance assessment/feedback but also VR-based vestibular-ocular motor and cognitive assessments.

### Limitations

This study is primarily a proof-of-concept demonstrating that measurements obtained from the IMU of a specific COTS head-mounted wearable (Glass) can provide quantitative balance measurements. These head-based measurements are comparable with waist-based measurements when following the NIH Standing Balance Toolbox protocol. This report only describes one quantitative balance measurement derived from accelerometry, NPL, although there are a variety of methods to preprocess these types of data [73]. A variety of subjective and objective assessments exist to both identify and characterize balance deficits [74]. A comprehensive characterization of measures obtainable from smartglasses against a clinical force plate system would provide a more thorough assessment of the concurrent validity of head-based measurement. Recently published pilot results from an elderly population using a force plate system support the potential for head-based measurement using COTS hardware in clinical assessment of balance [67].

Stances in the BAM protocol and NIH Standing Balance Toolbox were used in this study as a standard for preliminary comparison, given the availability of detailed methods and baseline data [31,38,39]. However, it has been previously suggested that as this protocol was not designed with the goal of concussion assessment, it may have limited use in this domain in comparison with other protocols. In one report, BAM was found not to effectively discriminate between healthy and concussed adolescents. Rather, expert scoring of the Balance Error Scoring System (BESS) protocol was able to identify patients from healthy participants with 60% sensitivity and 82% specificity [48]. The BESS protocol is similar to the BAM protocol with 6 conditions in total. However, in BESS, all conditions are performed with eyes closed and hands positioned on the hip, with 3 stances (feet together, single leg, and tandem stance) performed on both firm and foam surfaces. A modified BESS protocol, which eliminates the foam surface conditions, has been included as part of the SCAT sideline concussion evaluation since the second edition [75]. Although the modified BESS protocol may lack sensitivity, instrumenting the modified BESS with a waist-based inertial sensor led to superior diagnostic classification of recently concussed individuals compared with BESS alone, albeit in a relatively small sample size of 13 recently concussed individuals and 13 demographically matched controls [76]. In summary, future evaluations should consider whether other procedures are necessary, depending on study goals.

The study presented here is limited by its exclusive use of healthy subjects. Further research is necessary to determine whether measurements using a head-mounted device can detect deficits in postural sway related to specific medical conditions. Although the BAM protocol was unable to sufficiently discriminate concussed adolescents, postural sway as measured by waist-based BAM using this protocol was able to discriminate between persons with peripheral vestibular impairments and those without balance impairment [39]. In general, postural sway measurement alone currently lacks the sensitivity and specificity needed to confidently diagnose concussions. It is important to reiterate that our study goal was to demonstrate the feasibility of obtaining objective and quantitative measurements of postural sway with a head-mounted wearable. We hypothesize this would serve as only one component of a concussion assessment battery that could be automatically administered using COTS smartglasses as a platform.

Finally, although we determined that head-based measurement was generally consistent with waist-based measurement, head-based measurement might present additional challenges when administered outside carefully monitored conditions. For example, head-based measurement may be more sensitive to behavioral artifacts such as speech and shifting attention. It is important to mitigate these challenges by detecting and removing these artifacts to improve the internal validity of the assessment when used independently for clinical decision-making.

### Conclusions

The accelerometer built into Glass is sufficient to provide standing balance measurements comparable with commercial smartphones. Accelerometry measurements obtained from the head, including the NPL of AP sway as well as the total NPL magnitude, resulted in similar condition effects as those obtained from the waist in a healthy adult population. Head-based measurement of balance using smartglasses could serve as one component of a wearable, multifactorial concussion assessment that has integrated instruction and feedback. This approach could improve the objective assessment of concussion symptoms in high-risk activities, including contact sports and warfare, where current standards often rely on subjective methods, including symptom self-report by the injured. Smartglasses may provide a safer, lighter-weight, more portable, and more hygienic alternative to fully occlusive head-mounted wearables, while providing a similar range of assessments for concussion detection, including cognitive and vestibular-ocular motor screens. Further research is necessary to demonstrate the ability of smartglasses to detect balance dysfunction stemming from concussion.

## Abbreviations

ACC: accelerometer data
AP: anterior-posterior
BAM: balance accelerometer measure
BESS: Balance Error Scoring System
COTS: commercial off-the-shelf
FDA: Food and Drug Administration
ICC: intraclass correlation coefficient
IMU: inertial measurement unit
MACE: Military Acute Concussion Evaluation
mTBI: mild traumatic brain injury
NIH: National Institutes of Health
NPL: normalized path length
SCAT: Standardized Concussion Assessment Tool
VR: virtual reality
